# Establishing a Rodent Model of Ventricular Fibrillation Cardiac Arrest With Graded Histologic and Neurologic Damage With Different Cardiac Arrest Durations

**DOI:** 10.1097/SHK.0000000000001004

**Published:** 2018-07-13

**Authors:** Florian Ettl, Ingrid A.M. Magnet, Wolfgang Weihs, Alexandra-Maria Warenits, Daniel Grassmann, Michael Wagner, Ursula Teubenbacher, Sandra Högler, Fritz Sterz, Andreas Janata

**Affiliations:** ∗ Department of Emergency Medicine, Medical University of Vienna, Vienna, Austria; † Department of Pathobiology, University of Veterinary Medicine of Vienna, Vienna, Austria

**Keywords:** Cardiac arrest, cardiopulmonary resuscitation, histology, long-term outcome, rat, ventricular fibrillation

## Abstract

**Purpose:**

The aim of the study was to establish a ventricular fibrillation (VF) cardiac arrest (CA) resuscitation model with consistent neurologic and neuropathologic damage as potential therapeutic target.

**Methods:**

Prospectively randomized groups of experiments in two phases. In *phase 1* four groups of male Sprague–Dawley rats (n = 5) were resuscitated after 6 min VFCA with 2 and 6 min basic life support durations (BLS) with and without adrenaline. In *phase 2* the most promising group regarding return of spontaneous circulation (ROSC) and survival was compared with a group of 8 min CA. Resuscitability, neurologic deficit scores (NDS), and overall performance category (OPC) were assessed daily; histolopathology of the hippocampal CA1 region [hematoxylin and eosin- (viable neurons), Fluoro-Jade- (dying neurons), and Iba-1 immunostaining (microglial activation–semiquantitative)] on day 14.

**Results:**

Two minutes BLS and with adrenaline as most promising group of *phase 1* compared with an 8 min group in *phase 2* exhibited ROSC in 8 (80%) vs. 9 (82%) animals and survivors till day 14 in 7 (88%) (all OPC 1, NDS 0 ± 0) vs. 6 (67%) (5 OPC 1, 1 OPC 2, NDS 0.83 ± 2.4) animals. OPC and NDS were only significantly different at day 1 (OPC: *P* = 0.035; NDS: *P* = 0.003). Histopathologic results between groups were not significantly different; however, a smaller variance of extent of lesions was found in the 8 min group. Both CA durations caused graded neurologic, overall, such as histopathologic damage.

**Conclusions:**

This dynamic global ischemia model offers the possibility to evaluate further cognitive and novel neuroprotective therapy testing after CA.

## INTRODUCTION

Sudden cardiac arrest (CA) is a worldwide burden and leading cause of death. After primary successful cardiopulmonary resuscitation (CPR) survival with severe neurological disability is often the consequence (1). Despite decades of research and numerous updates of resuscitation guidelines, survival and outcome remain poor (2–4). The pathophysiology during CA is multifactorial and complex (5). Obviously randomized human trials are rare.

These facts stress the importance of animal models that offer the possibility to search for and test new treatment options with the potential to improve outcome. Therefore, experiments mimicking clinically realistic long-term outcome studies are necessary. The Vienna Resuscitation Research Group has gained expertise in resuscitation research with a pig model (6–9). However, large animal models are too elaborative and expensive for basic research with high number of cases needed. In more cost-effective rodent models, a wide variety of molecular and immune-histochemical investigational tools are available. Furthermore, rats offer the possibility for the whole spectrum of resuscitation research including extracorporeal life support (ECLS) (10).

Reproducibility, stability, and clinical realism of the model were our main focus in this study. Different rat models of VF CA have been used by various groups. Two main approaches became evident during literature search, differing in the use of adrenaline and the duration of chest compressions before the first shock (10–15). One successful, extensively published approach uses a prolonged period of chest compressions and no epinephrine, adding a longer low-flow phase to the cardiac arrest (10, 14, 15). Another approach used over a long time in multiple studies uses epinephrine and defibrillations after a short CPR period (11–13). Epinephrine has been shown to exert adverse effects on the rat heart (16), data on brain perfusion are conflicting (17, 18). Furthermore, the duration of arrest might affect the outcome in these two approaches, e.g., a longer arrest might need epinephrine to be resuscitated successfully. By using the same setup for both approaches, the same experimenters and same rat strains, we hoped to better understand how the model choice affects outcome, and thereby which model might be suited to answer which question, and offers the greatest reproducibility.

It has never been tested how theses paradigms compare in terms of resuscitability, long-term survival, and histopathology. The aim of this study was to use two well-established rat VF models to determine how variables like no flow, low flow, and adrenaline affect outcome. We thereby hoped to better understand the effects of CPR, adrenaline and arrest time on our experiments, and perhaps set a firm foundation also for future researchers who struggle to choose the optimal model for their scientific questions.

## METHODS

**Fig. 1:**
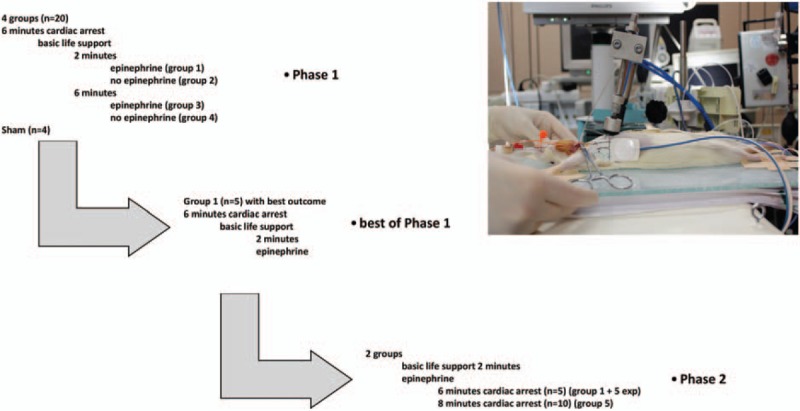
Study design and experimental setup.

To save animals and resources the study was planned in two phases such as assessing in *phase 1* in a randomized sequence 4 groups (each n = 5) of 6 min CA with different durations (2 and 6 min) of “basic life support” with and without the use of adrenaline during resuscitation before the first defibrillation attempt. Thereafter in *phase 2* the five animals of the group with best outcome of *phase 1* were restocked with five additional animals and compared in a randomized sequence with a group of 8 min CA animals (n = 10). Sham animals (n = 4) underwent the same surgical procedure without CA and resuscitation (Fig. 1).

*Phase 1*: Four groups of 6 min CA with different durations (2 and 6 min) of “basic life support” with and without adrenaline. *Phase 2*: The group with best outcome of *phase 1* was compared with a group of 8 min cardiac arrest.

The animal investigation committee of the Medical University of Vienna approved the experimental protocol (https://www.meduniwien.ac.at/hp/dbf/services/ethikkommission/GZ:66.009/0064-II/3b/2011) in compliance with current Austrian and European legal requirements (Directive 2010/63/EU) and good laboratory practice guidelines (19).

A total of 49 adult male Sprague–Dawley rats (350 ± 25 g; Himberg, Austria) were brought to the laboratory 14 days before the experiment, maintained on 12:12 h light/dark circle with *ad libitum* access to water and feed and adapted to the unfamiliar environment. For surgery sedation was induced via the administration of sevoflurane 6% for 4 min in a box. The rats were intubated with an adapted cannula (14GA Venflon BD Luer-Lok, Helsingborg, Sweden) and ventilated volume-controlled with 65/min, 7 mL/kg bodyweight (BW) and 0.3 FiO_2_ (Havard Inspira advanced safety ventilator, volume controlled, MA1 55-7058, Holliston, Mass). To maintain anesthesia buprenorphine 50 μg/kg BW was given subcutaneously (s.c.) after intubation and sevoflurane 3.5% was further administered via the ventilator. Temperature probes (General Purpose Sensor 9F, Mon-a-therm, A Mallinckrodt Company, Mexico) were placed in the esophagus (Tes) and rectum (Trec). The temperature level was maintained at 37 ± 0.2°C with an operating table for small animals (Medax GmbH & Co, Neumünster, Germany). Two catheters (Argyle Polyurethane Umbilical Vessel Catheter; 2.5 Fr, Convidien, Mansfield, Mass) were inserted over the left femoral vein and left femoral artery for hemodynamic monitoring, blood sampling, and for the administration of medications. Electrocardiogram (ECG), mean arterial pressure (MAP), central venous pressure (CVP), and EtCO_2_ were continuously monitored and recorded (Philips IntelliVue MP70 Patient Monitor, Philips Healthcare, Andover, Mass). A neonatal pacing catheter (Vygon GmbH & Co Bi-Pacing-ball 3 Fr, Aachen, Germany) was inserted 5 cm into the right jugular vein toward the right heart for the induction of VF. After surgery, the rat was transferred and fixed tightly to allow efficient thorax compression. Cut adhesive infant defibrillation paddles (Multifunction Infant Electrode Pads Plus, Philips) were stitched to the shaven right and left lateral thorax and connected to a biphasic manual defibrillator (HeartStart MRx ALS Monitor/Defibrillator, Philips).

### Experimental protocol

**Fig. 2:**
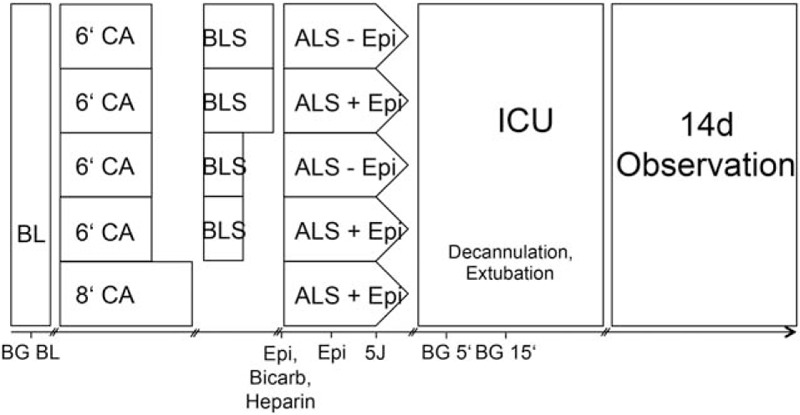
Timelines of experimental protocols.

An arterial blood gas analysis was performed 5 min before the induction of CA (Fig. 2). Inhalative anesthesia was discontinued 1.5 min before start of VF induction. Mechanical ventilation was stopped and VFCA was induced by a 50 Hz alternating electrical current of maximum 12 mA for 2 min via the pacing catheter. Once CA was confirmed by a drop in MAP and loss of pulsatility in the femoral arterial line, the timer was started. If spontaneous conversion in sinus rhythm occurred, additional 30 s impulses were delivered. After 6 or 8 min of untreated VFCA, resuscitation was started with mechanical chest compressions (200/min) delivered with a pneumatic chest compression device (Streubel Automation, Grampersdorf, Germany) and mechanical ventilation (20/min, 7 mL/kg BW, 1.0 FiO_2_). The compressions were applied approximately 1 cm cranial of the lower sternal rim and the compression depth was adapted according to the monitored MAP at 60 mmHg. Depending on the group assignment rats were defibrillated (2 times with 5 J, biphasic) after 2 or 6 min of CPR and defibrillation attempts were repeated every 2 min. Bicarbonate 1 mmol/kg was given i.v. 15 s before the start of CPR. In the corresponding groups adrenaline 20 μg/kg BW or saline was administered with bicarbonate and continued with 10 μg/kg BW 60 s after start of CPR, and repeated every 2 min during CPR. If restoration of spontaneous circulation (ROSC) was not achieved after five double shocks the experiment was terminated. The investigators were blinded to the adrenaline/nonadrenaline groups but not to the duration of cardiac arrest or CPR until first defibrillation attempt.

*Phase 1:* Four groups (each n = 5); cardiac arrest (CA, 6 min); basic life support (BLS, 2 and 6 min); adrenaline (Epi, with and without) during resuscitation (ALS); defibrillation (J). *Phase 2:* Group with best outcome cardiac arrest (CA, 6 min) compared with a group cardiac arrest (CA, 8 min) plus adrenaline (Epi) (two groups, each n = 10). BL, base line; BG, blood gas; Bicarb, NaHCO3; ICU, intensive care.

### Postresuscitation care

After ROSC, the ventilation rate was increased to 65/min and further adaptions of the ventilation parameter were performed according to the arterial blood gas analyses 5 and 15 min after ROSC. Given the comatose status of the rats after reaching ROSC, the inhalative anesthesia was not resumed. If ROSC was sustained (>20 min MAP >50 mmHg), the catheters were removed and the animals weaned from mechanical ventilation. Hemodynamic stabilization was only provided with crystalloid fluid boles, no further catecholamines were administered nor where further resuscitation attempts started. The animals were provided with oxygen and heating lamps to keep them at 37 ± 0.5°C and were allowed free access to food and water. The rats were evaluated daily and received subcutaneous fluid (10 mL s.c. daily) if they did not drink adequately. Analgesia was maintained with buprenorphine 24 μg/kg BW s.c. as long as signs of pain were observed. As soon as obvious normal recovery was achieved, animals were kept in groups of four in a cage. If animals did not recover appropriately, the protocol was terminated earlier and the animals were euthanized with a sevoflurane and buprenorphine overdose.

Neurologic function was assessed daily by an investigator blinded to the study group, using a neurologic deficit score (NDS) (0% = normal, 100% = dead) (20) and an overall performance category score (OPC) (1 = normal; 2 = slight disability; 3 = severe disability; 4 = comatose; 5 = dead) (21, 22). On day 14 after assessment of final OPC and NDS, rats were euthanized with a sevoflurane overdose and perfused with normal saline followed by formalin 10% for histologic evaluation.

### Histopathology

Histologic evaluation was performed by an experienced veterinary pathologist blinded to the treatment groups. Brains were fixed in buffered formaldehyde solution 7.5% and embedded in paraffin wax. Coronary sections of 5 μm thickness were cut at bregma 3.5 to 4.0 to depict the hippocampal CA1 region and used for quantification of lesions. Furthermore, coronary sections including striatum, cerebral cortex, and cerebellum were cut. Sections were stained with hematoxylin and eosin (H&E) and with Fluoro-Jade B (FJB) (Merck Millipore, Darmstadt, Germany) according to manufacturer's instructions. In *H&E-staining* viable neurons with a visible nucleolus were counted in two 250 μm sectors of the medial and lateral CA1 region of the hippocampus at 200× magnification. H&E-stained sections were furthermore used for overall assessment of ischemic neuronal damage in the CA1 region. In *FJB-stained* sections fluorescent dying neurons were counted in a similar fashion as the viable neurons in H&E-staining. For detection of microglial cells in the CA1 region with *Iba-1 immunostaining* the primary antibody against ionized calcium-binding adaptor molecule 1(Iba1) (polyclonal anti-rabbit, dilution 1:80,000; Wako Chemicals GmbH, Neuss, Germany) was applied to the brain using autostainer 360 (Lab Vision, Thermo Fisher Scientific, Bonn, Germany). The detection system UltraVision LP, HRP Polymer (Lab Vision, Thermo Fisher Scientific, Fremont, Calif) was used according to manufacturer's instructions. DAB (3,3’-diaminobenzidine) was applied as chromogen (Lab Vision) and counterstaining was performed with Mayer's hematoxylin (Lab Vision). For antigen-retrieval sections were boiled in a microwave oven (700 W) twice for 5 min in 0.01 M citrate buffer (pH 6). The staining intensity of microglial cells was assessed semiquantitatively on a 5-point scale, ranging from normal mild staining (0 points) to very intense staining (4 points) of microglia.

### Statistical analysis

Continuous data that were normally distributed are reported as mean and SD; data that were not normally distributed are reported as median and 25th/75th percentile. Group comparisons were made with one-way analysis of variance and the Student–Newman–Keuls test for pair-wise analysis, or the Kruskal–Wallis test, as appropriate. Categorical variables are reported as counts. Group comparisons were made with the chi-square test or Fisher exact test, as appropriate. The significance level was a two-sided *P* value of <0.05. All calculations were performed with IBM Statistics for Mac 22 (SPSS, Chicago, Ill).

## RESULTS

Of 49 rats in this study, 9 were excluded due to failures during surgery or during the experimental procedures. The sham group consisted of four rats.

Outcome during *phase 1* was as follows (Table 1): in the study group “2 min CPR without adrenaline,” no rat had ROSC. In the group “6 min CPR without adrenaline,” 1 rat had ROSC (20%) but died during the first night. In the group “2 min CPR with adrenaline”, 4 rats had ROSC (80%), all of which survived to 14 days with OPC 1 and an NDS of 0 ± 0. In the group “6 min CPR with adrenaline,” 2 rats had ROSC (40%) and survived to day 14 with OPC 1 and NDS 5—both rats showed motoric deficits on the travel ledge test. For *phase 2* of the study, all rats were resuscitated with 2 min CPR until the first defibrillation attempt with the use of adrenaline, the most promising method of *phase 1*.

**Table 1:** Restoration of spontaneous circulation (ROSC) and survival till endpoint on day 14

Protocol	Cardiac arrest	6 min				8 min
	“Basic life support”	6 min		2 min		2 min
	Adrenaline	No	Yes	No	Yes	Yes
Outcome	No ROSC	••••	•••	•••••	••	••
	ROSC	•	••		••••••••	•••••••••
	Survival		••		•••••••	••••••

Each dot represents one rat.

Outcome data for *phase 2* are presented in Tables 1 and 4. ROSC rate was 80% for 6 min CA and 82% for 8 min CA. We compared the neurologic and histologic outcome of the 7 (88%) survivors of the 6 min CA group to the 6 (67%) survivors of the 8 min CA group. Baseline values of these two groups are presented in Table 2 without significant differences between groups and arterial blood gas values 5 and 15 min after ROSC is presented in Table 3. Neurological outcome (OPC and NDS) is presented in Table 4. A significant difference between the 6 and 8 min CA group could only be shown on day 1 (OPC, *P* = 0.035, NDS, *P* = 0.002) and equalized from day 2 on.

**Table 2:** Physiologic baseline values and resuscitation variables of *phase 2* experiments without significant differences between groups

		Cardiac arrest	
		6 min (n = 7)	8 min (n = 6)
	Sham (n = 4)	Advanced life support and adrenaline	
Weight (g)	376 ± 11	366 ± 17	350 ± 16
Surgery time (min)	99.3 ± 46.7	78.6 ± 11.8	83.7 ± 11.9
Heartrate (/min)	366 ± 62	383 ± 30	375 ± 25
Temperature (°C)	36.9 ± 0.1	37.1 ± 0.1	37.1 ± 0.1
MAP (mmHg)	98 ± 22	110 ± 10	114 ± 12
pH	7.42 ± 0.02	7.41 ± 0.04	7.39 ± 0.02
paCO_2_ (mmHg)	40.9 ± 3.2	40.1 ± 6.5	40.9 ± 3.8
paO_2_ (mmHg)	136 ± 31	133 ± 34	104 ± 10
Hb (g/dL)	14.1 ± 0.5	14.4 ± 1.2	14.2 ± 1.1
K (mmol/L)	4.2 ± 0.7	4.2 ± 0.6	4.4 ± 0.5
Glucose (mg/dL)	181 ± 31	183 ± 42	162 ± 20
Lactate (mmol/L)	1.8 ± 0.3	1.4 ± 0.6	1.1 ± 0.1
CPR time (min)		2.6 ± 1.0	2.7 ± 1.0
Defibrillations (n)		1.7 ± 1.0	2.3 ± 1.0
Epinephrine (mcg)		45.7 ± 9.8	46.7 ± 10.3

CPR, cardiopulmonary resuscitation; Hb, haemoglobin; K, potassium; MAP, mean arterial pressure; paCO_2_, arterial carbon dioxide partial pressure; paO_2_, arterial oxygen partial pressure. Data are presented as mean ± SD.

**Table 3:** Arterial blood gas analyses of phase 2 experiments taken (a) 5 min and (b) 15 min after ROSC

(a)				
		Cardiac arrest		
	Sham (n = 4)	6 min (n = 7)	8 min (n = 6)	*P*
		Advanced life support and adrenaline		
pH	7.42 ± 0.24	7.09 ± 0.05	7.03 ± 0.06	0.104
paCO_2_ (mmHg)	40 ± 3	53 ± 8	54 ± 10	0.861
paO_2_ (mmHg)	130 ± 20	265 ± 58	291 ± 89	0.546
Hb (g/dL)	14 ± 0.2	15 ± 1	15 ± 1	0.930
K (mmol/L)	4.2 ± 0.4	*4.4 ±* *0.2*	*5.7 ±* *0.7*	*0.001*
Glucose (mg/dL)	168 ± 21	334 ± 26	346 ± 42	0.544
Lactate (mmol/L)	1.4 ± 0.2	*10.5 ±* *1.2*	*12.1 ±* *0.9*	*0.016*

(b)				
	Sham (n = 4)	Cardiac arrest		
		6 min (n = 7)	8 min (n = 6)	*P*
		Advanced life support and adrenaline		
pH	7.41 ± 0.32	7.20 ± 0.11	7.12 ± 0.73	0.136
paCO_2_ (mmHg)	41 ± 3	48 ± 7	52 ± 5	0.344
paO_2_ (mmHg)	130 ± 21	273 ± 93	310 ± 60	0.424
Hb (g/dL)	14 ± 1	15 ± 1	15 ± 1	0.554
K (mmol/L)	4.2 ± 0.6	*3.4 ±* *0.4*	*3.9 ±* *0.3*	*0.018*
Glucose (mg/dL)	172 ± 10	295 ± 39	302 ± 31	0.739
Lactate (mmol/L)	1.5 ± 0.4	6.9 ± 1.7	8.3 ± 2.0	0.213

Hb, haemoglobin; K, potassium; paCO_2_, arterial carbon dioxide partial pressure; paO_2_, arterial oxygen partial pressure. Significant differences between the two cardiac arrest groups and corresponding *P* values are marked italic. Data are presented as mean ± standard deviation.

**Table 4:** Outcome in terms of final overall performance categories (OPC 1: best, 5: worst) and neurologic deficit scores (NDS 0%–100%, mean ± SD) of phase 2 experiments at day 1 and day 14 after return of spontaneous circulation (*P* < 0.05)

		Cardiac arrest			
	Sham	6 min	8 min	6 min	8 min
		Advanced life support and adrenaline			
		Day 1		Day 14	
OPC 1	••••	••••••	•	•••••••	•••••
OPC 2		•	•••••		•
OPC 3					
OPC 4					
OPC 5					
NDS	0.25 ± 0.5	3.83 ± 3.19	12.83 ± 4.49	0 ± 0	0.83 ± 2.04

Each dot represents one rat.

### Histology results

**Fig. 3:**
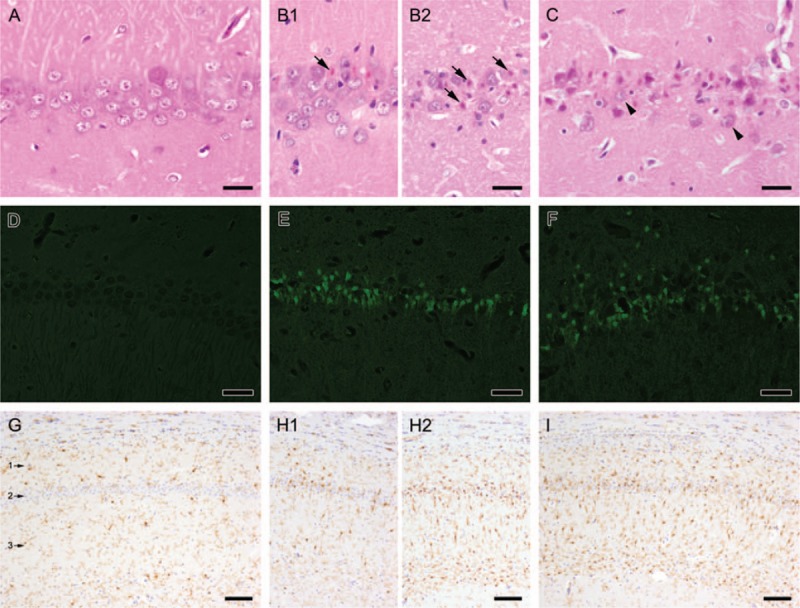
Representative sections of the hippocampal CA 1 region after 0, 6, and 8 min of cardiac arrest (CA).

H&E sections showed consistent lesions in the hippocampal CA1 region of animals subjected to VFCA. In striatum, cerebral cortex, and cerebellum, no lesions were detectable. Representative images of the hippocampal CA1 region in all staining methods are shown in Figure 3. Results of cell counts of viable neurons (*H&E-staining*) and dying neurons (*FJB-staining*) and of the semiquantitative evaluation (*Iba-1 immunostaining*) of microglial activation are presented in Table 5. In all staining methods, there were statistically significant differences between the sham group and both CA groups, but no significant differences were detectable between 6 and 8 min CA group. There was a trend toward more consistent severe lesions in the 8 min CA group, whereas in the 6 min CA group a wide variance of extent of lesions was present. In *H&E-staining* a lot of degenerated neurons with hypereosinophilic cytoplasm and dark shrunken nuclei were detectable in all animals of the 8 min CA group in the CA1 region of the hippocampus. These neurons were positive in *FJB-staining* as well. In the 6 min CA group three animals showed severe lesions like the 8 min CA group (Fig. 3B2), whereas four animals showed only mild-to-moderate lesions with a lot of remaining viable neurons (Fig. 3B1). Sham animals did not show any degenerated neurons in *H&E-staining* or fluorescent neurons in *FJB-staining*. For reference, though not statistically evaluated, the two surviving 6 min CA and 6 min CPR with adrenaline rats from *phase 1* of the study showed damage in the CA1 region of the hippocampus with a viable neuron count of 9 and 18 in H&E-staining and 56 and 53 dying neurons in FJB-staining. Histologic evaluation of the other study groups of *phase 1* was not possible because there were no survivors. *Iba-1 immunostaining* with immunohistochemistry using Iba1-antibody revealed moderate-to-severe microglial response in both CA groups. In the pyramidal layer and molecular layer of the hippocampal CA1 region a lot of activated microglial cells with short and thick processes and rod-shaped soma were detectable in all animals of the 8 min CA group. Similar microglial reaction was detectable in animals with severe neuronal lesions in the 6 min CA group. In contrast animals with mild-to-moderate neuronal lesions in the 6 min CA group showed only moderate microglial activation in the hippocampal CA1 region. In sham animals only resting microglia with long and fine processes and very little cytoplasm were present in this region.

(A–C) H&E-staining , bar = 40 μm; (A) only viable neurons in sham animal; (B1) few degenerated neurons (arrow) in mildly affected 6 min animal; (B2) many degenerated neurons (arrows) in severely affected 6 min animal; (C) only few viable neurons (arrowheads) present in 8 min animal; (D) no degenerated fluorescent neurons in sham animal; (E, F) FluoroJadeB-staining, bar = 50 μm: many degenerated fluorescent (bright green) neurons in both CA groups; (G–I) Iba1-immunohistochemistry, bar = 150 μm; (G) few resting microglia (brown) in oriens (1) and radiatum (3) layer of the hippocampus in sham animal; (H1) moderately activated microglia, especially in oriens (1) and pyramidal cell (2) layer in mildly affected 6 min animal; (H2) many activated microglia in oriens (1), pyramidal cell (2), and radiatum (3) layer in severely affected 6 min animal; (I) similar activation of microglia as in H2 in 8 min animal.

**Table 5:** Neuropathologic damage of phase 2 experiments at 14 days after cardiac arrest

		Cardiac arrest	
	Sham (n = 4)	6 min (n = 7)	8 min (n = 6)
		Advanced life support and adrenaline	
Haematoxylin and eosin (counts of viable neurons)	58.75 ± 6.08	27.86 ± 14.70	16.67 ± 4.31
Fluoro-Jade (counts of dying neurons)	0.00 ± 0.00	45.14 ± 19.23	62.67 ± 9.14
Iba-1 immunostaining (semiquantitative–microglial activation)	0 (0–0)	3 (3–3)	3 (3–4)

Normally distributed values: mean ± SD; not normally distributed values: median (25–75th percentile).

## DISCUSSION

The 6 min CA group of *phase 1* with 2 min “basic life support” and adrenaline during advanced life support turned out to be the most promising group with the highest ROSC and survival rate. Therefore, these animals were compared in a block randomized design in *phase 2* with an 8 min CA group with 2 min “basic life support” and adrenaline during advanced life support. These two groups proved to produce consistent damage and provide the basis for further resuscitation studies.

Despite given literature, resuscitation without adrenaline as well a prolonged “basic life support” period aiming for more clinical realism were not feasible in our setting (11, 23–25). Two minutes of CPR without adrenaline did not sufficiently perfuse the heart before defibrillation to produce ROSC. After 6 min of CPR without adrenaline before defibrillation at least one animal achieved ROSC but did not survive until the final endpoint on day 14. We initially expected that the prolongation of the “basic life support” period and thereby uninterrupted chest compressions to adequately perfuse the heart before the first shock would allow us to omit the use of adrenaline to achieve ROSC and thereby leading to a better neurological outcome (26). As there were no survivors in the nonadrenaline groups, we unfortunately are not able to compare neurologic outcome between adrenaline and nonadrenaline rats. Six minutes of CPR together with the use of adrenaline during the following advanced life support resulted in a ROSC and survival rate under 50%. Although clinically more realistic, a model with a low survival rate was not feasible for us. To our knowledge this is the first study of a head-to-head comparison of these two concepts of rat resuscitation after VF CA—prolonged “basic life support” or the use of adrenaline.

In *phase 2*, the longer CA of 8 min expectedly led to higher lactate and potassium values in the first arterial blood gas analyses after ROSC. Both groups (6 and 8 min CA) had a very good neurological and histological outcome and differed significantly only in the neurological outcome on day 1. This difference equalized from day 2 on. On day 14 nearly all animals fully recovered neurological with a tendency for more deficits in the group with the longer insult.

The applicability of our results for the human patient is limited due to general rodent and animal model limitations. In our initially healthy rats the CA was the result of electrical current application and not of underlying pathologies. In contrary to the human patient rats tend to convert spontaneously back in sinus rhythm. Also, we experienced that rodents tend to recover to normal neurologic function of gross examination if they do not die in the early postresuscitation phase. The rodent model is suitable as basic resuscitation research model offering the possibility to further get to know the underlying pathophysiology and the testing of future treatment strategies. These experiments are the base for further studies in the resuscitation research cascade with large animal and following human studies.

Temperature control is easily achieved in rodents with surface cooling. Against given resuscitation guidelines we did not cool down the rats to mild therapeutic hypothermia after ROSC in this study. This intervention and the comparison of different temperature groups are planned for future studies. Furthermore, we applied sodium bicarbonate before resuscitation efforts based on the given experiences with the Pittsburgh model (10). This also cannot be found in the given human resuscitation guidelines but was necessary in the rodent model to successfully reach ROSC.

The maximal arrest duration for VFCA with good resuscitability reached with conventional CPR has not been assessed till today and needs to be further investigated. This is necessary to have the possibility for studying the full potential of further treatment strategies. This rodent model offers now the possibility to perform studies with higher case number necessary like search for a potential dose effect of hypothermia or the possibility for the induction of a preservative state in deep hypothermia and its benefits. Also, other resuscitation techniques like the use of ECLS with a high number of unanswered questions like optimal bypass settings, priming solutions, and so on are possible targets for further studies.

We successfully established a rodent 6 and 8 min VFCA arrest model with consistent neurological and histological damage and good resuscitability. Most survivors achieved full recovery of neurological function reflected in OPC and NDS. The high ROSC rate and good neurological and histological outcome after 14 days survival may allow further prolongation of the CA duration in future studies. In our model resuscitation without adrenaline as well as prolongation of basic life support lead to worsened outcome and did not appear feasible for upcoming experimental protocols. Our model will be the basis for future interventional studies with prolonged CA. It allows further investigation of the pathophysiology of CA and resuscitation and will be compared with our planed ECLS model.
